# A tool for rapid, automated characterization of population epigenomics in plants

**DOI:** 10.1038/s41598-023-38356-7

**Published:** 2023-08-17

**Authors:** Jack M. Colicchio, Cynthia L. Amstutz, Nelson Garcia, Keerthana N. Prabhu, Thomas M. Cairns, Melis Akman, Thomas Gottilla, Twyla Gollery, Shawn L. Stricklin, Travis S. Bayer

**Affiliations:** Sound Agriculture Company, Emeryville, CA USA

**Keywords:** Epigenetics, DNA methylation, Agricultural genetics, Computational biology and bioinformatics, Plant sciences

## Abstract

Epigenetic variation in plant populations is an important factor in determining phenotype and adaptation to the environment. However, while advances have been made in the molecular and computational methods to analyze the methylation status of a given sample of DNA, tools to profile and compare the methylomes of multiple individual plants or groups of plants at high resolution and low cost are lacking. Here, we describe a computational approach and R package *(sounDMR*) that leverages the benefits of long read nanopore sequencing to enable robust identification of differential methylation from complex experimental designs, as well as assess the variability within treatment groups and identify individual plants of interest. We demonstrate the utility of this approach by profiling a population of *Arabidopsis thaliana* exposed to a demethylating agent and identify genomic regions of high epigenetic variability between individuals. Given the low cost of nanopore sequencing devices and the ease of sample preparation, these results show that high resolution epigenetic profiling of plant populations can be made more broadly accessible in plant breeding and biotechnology.

## Introduction

Tremendous progress over the past decades in genome sequencing technologies and computational genomics have transformed our understanding of biology, yet it is becoming clear that while a great deal of phenotypic variation can be explained by genetic differences, the epigenetic markers associated with DNA, such as methylation of cytosine residues, can also have dynamic effects on health, development, and ecology^1–4^. These epigenetic marks are not only inherited between generations^5–7^, but can also be directly modified by an organism's environment within a single generation^8–10^, conferring unique properties to this information^11^. Epigenetic analysis promises insight into a diverse range of fields^12–14^, but progress has been limited by the difficulty of reading the epigenetic state of DNA sequences. Advances in sequencing technology (such as Pacific Biosciences^15,16^ and Oxford Nanopore^17–21^) have enabled base-resolution 5mC methylation calling directly from DNA (as well as other base modifications) without the need for additional chemical treatment. Here, we describe statistical and computational methods designed to work with the epigenetic readouts from ONT sequencing technologies, with the aim of democratizing epigenetic research and better illuminating the complex relationships between the epigenome, the environment, and development.

Bisulfite treatment of DNA followed by Illumina sequencing (WGBS) has been considered the gold standard for methylome studies requiring quantitative estimates of DNA 5mC methylation at base-pair resolution. However, recent work has demonstrated that similar levels of accuracy can be achieved using Oxford Nanopore Technology (ONT) sequencing and computational advances now enable accurate methylation calling even in non-CG contexts^20^, which is a vital epigenetic mechanism in plant transposon regulation. Nanopore sequencing offers several advantages in both accessibility and power. Unlike WGBS, methylome analysis with nanopore devices does not require any DNA treatment prior to library preparation, and whole genome methylome sequencing can be performed with a 200 USD device. The ability to perform real-time sequencing and adaptive sampling allows for targeted methylome analyses of specific regions of the genome at high coverage, and the use of long-reads enables mapping to complex regions of the genome, as well as methylome phasing. Thus, the main limitations of WGBS that are overcome through ONT are the capacity to perform methylome analysis without specialized library preparations and using a very inexpensive sequencing device, the ability to call methylation in difficult to map or repetitive regions, and the ability to perform methylome phasing.

These features allow for low cost methylome sequencing of relatively large numbers of individuals, but require the development of tools that play to these strengths. Within the past few years, a number of tools have been developed that have begun to leverage these capabilities, even enabling phased methylome analysis^21–23^, and visualization packages have been created that make use of the long reads sequenced on the ONT platform^24,25^. Here, we extend this toolkit by presenting methods aimed at expediting a broad range of methylome analyses that work to the strengths of nanopore sequencing and fill gaps in the current suite of tools. With the release of tools like pycoMeth^26^ the ability to detect differentially methylated regions (DMRs) using ONT data has taken great strides, but here we provide a useful alternative to further elucidate differences in methylation across sequenced samples. In particular *sounDMR* handles situations with complex experimental designs using a mixed modeling approach, simplifies methylome analysis of not just CG but also CHG and CHH methylation, and integrates a changepoint detection and bootstrapping approach to confidently identify regions where either mean or variance of methylation has shifted. It is worth noting that many of the new methods described here have been inspired by the array of tools available for WGBS analysis (see recent reviews^27,28^).

**Figure 1:**
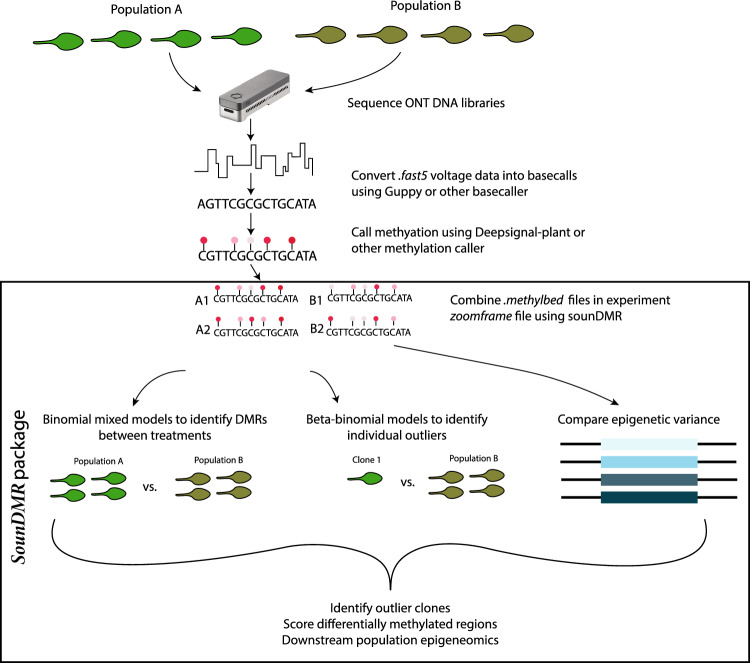
Experimental workflow for population epigenomic profiling using *sounDMR*. Multiplexing along with adaptive sampling on Oxford Nanopore devices allows for the relatively rapid and inexpensive sequencing of large numbers of individuals (shown here 8 individuals, that would be barcoded separately and multiplexed for sequencing, however this number would vary depending on the user's experimental design). Using tools already available we perform basecalling and methylation calling from this sequencing data. After individual methylomes have been established, our new package *sounDMR* combines these into a single project file and allows for statistical analyses using a flexible mixed modeling framework to compare methylation between groups of individuals, or specific clones of interest. Additionally, this pipeline makes it easy to estimate within group epigenetic variance at a per-cytosine level, enabling hyper-variable epigenomic regions to be identified. This data is all output into a unified output data frame that enables downstream analysis into population epigenomic patterns.

To demonstrate this approach, we sequenced *Arabidopsis thaliana* plants exposed to various concentrations of a known 5mC demethylating agent, zebularine^29^. While previous work has demonstrated that increasing concentrations of zebularine decreases DNA methylation^30^, the consistency of the demethylation effects across plants is largely unknown. We used nanopore adaptive sampling to sequence *A. thaliana* individuals, and then use *DeepSignal-plant*^20^ to call methylation frequencies. The R package presented here, *sounDMR*, simplifies the creation of an easy to analyze methylome experiment file. From the methylome experiment file, a variety of mixed models can be applied, as well as investigation of group shifts in methylation, quantification of the variability of the methylome in different regions of the genome, and the identification of specific individuals that show extreme methylation patterns in a given region (Fig. 1). Finally, we integrate changepoint analysis to break the genome into contiguous regions of similar baseline methylation, or regions with similar shifts in methylation in response to a factor of interest in order to detect differentially methylated regions (DMRs). These approaches provide a straightforward toolset that brings together a variety of methods useful in the analysis of DNA methylation, and create a simple data structure that increases the ease in which ONT sequencing can be turned into novel epigenetic insight. Using zebularine treated *Arabidopsis thaliana*, here we provide examples of the types of analyses that can be performed using this methodology.

## Results

### Creation and sequencing of epigenetically diverse plant populations

To induce epigenetic variation, *A. thaliana* seeds were treated with three different concentrations of zebularine. We will describe these three different zebularine concentration groups, and our water and DMSO controls as “populations” throughout the remainder of this paper. Plants displayed stunted vegetative growth phenotype that inversely correlated with the supplied concentration of zebularine (Fig. S1). While the individuals treated with 100 µM zebularine were severely stunted, the individuals treated with 50 and 25 µM zebularine were moderately and mildly stunted, respectively, compared to the water and DMSO controls.

ONT adaptive sampling of the 32 *A. thaliana*  individuals across the 426 genes (and regions within 10kb of these genes) included in our adaptive sampling panel (Table S2) were basecalled using *Guppy* and had 5mC  methylation quantified using *DeepSignal-Plant* and *Megalodon*. A total of ~ 3.23 million cytosines were analyzed through the pipeline for all the samples that had an average read depth of 25.27 and a median read depth of 17 around the target region (Table S1). In order to evaluate the accuracy of the model, we compared our data with publicly available WGBS data^6,31^ from NCBI (Note S1, Fig. S2). We found that mean methylation levels across all three sequence contexts were more similar between *DeepSignal-Plant*^20^ and WGBS data than *Megalodon* and WGBS, and that basewise correlations were higher for *DeepSignal-Plant* (Correlation: CG: 0.92 vs. 0.88, CHG: 0.86 vs. 0.56, CHH 0.75 vs. 0.32). For this reason we opted to use *DeepSignal-Plant* for methylation calls; however, our package is compatible with “bedMethyl” files generated from either model and can be adapted to future methyl-calling methods including the recently released *Dorado* basecaller and modified basecaller by ONT (Fig. 1).

### SounDMR detects changes to global methylation

**Figure 2:**
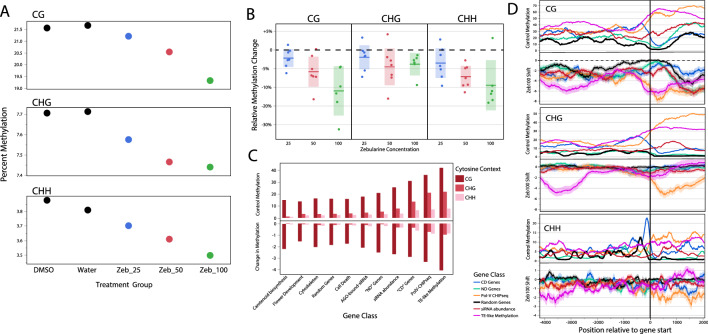
ONT data captures methylation concentration and gene class dependent methylation decreases in response to zebularine. (**A**) Baseline methylation across all sequenced cytosines decreases as zebularine concentrations increase from 25 to 100 μΜ. Comparisons between all zebularine groups are significant (*p* < 0.01) with the exception of Zeb_50 vs. Zeb_100 for CHG methylation. (**B**) Individual to individual variation in the global shift in methylation between zebularine treated plants and controls. Each point reflects a single *Arabidopsis thaliana* plant. (**C**) Baseline methylation in the 5 kb region up-stream of a gene and the shift in methylation in response to zebularine (Zeb_100 treatment) varies substantially across our different classes of genes (Table S2). (**D**) Spatial patterns of baseline and shifts in methylation further exemplify the variability of epigenetic responses between gene classes.

Across our adaptive sampling panel, we confirmed previous findings that zebularine induces 5mC demethylation in a concentration dependent manner (Fig. 2A). Additionally, we confirmed that while CG and CHH do not appear to reach a saturation point in demethylation at 100 µM^30^, there is no significant decrease in methylation in CHG between 50 and 100 µM. Next, we analyzed individual to individual variation for methylation within our adaptive sampling region and found that there was a great deal of variability for the extent of decrease in methylation imparted by the treatment (Fig. 2B). For instance, within the 100 µM treatment group, we found that individuals ranged from having 5–32% reductions in CG methylation relative to controls. In order to determine how treatment effected within-group methylation variability, we calculated per site variance within each treatment group and found that, relative to water controls, there was a 31% (25 µM) to 55% (50 µM) increase in variance of cytosine methylation in our treated groups. Interestingly, our DMSO control group also had more variable methylomes, suggesting that this epigenetic variability is induced at least in part by DMSO in addition to zebularine.

### Classes of genes differ in baseline methylation and response to zebularine

We chose 426 genes that represented a wide spread of functional categories for adaptive sampling including those that are known to be methylated through the association with RdDM machinery (Table S2, Data S1). As expected, these gene classes varied dramatically in baseline methylation in all three sequence contexts both within protein coding sequences (*p* < 0.0001 for all contexts) as well as in the 0–5 kb upstream promoter region (*p* < 0.0001 for all contexts, Fig. 2C). The proportion of variance explained by gene category varied across cytosine contexts and gene region, with promoter region methylation only having between 8 (CHH) and 13% (CHG) of variance explained by gene classification, while coding sequence variance had 29% (CHH) to 44% (CHG) of variance explained by gene class. One particularly striking pattern was seen within the “CD” class of genes, that were previously found to have methylation restored after the reintroduction of *NRPD1* into the genetic background (Table S2), and tend to have significantly higher promoter CG, CHG, and CHH methylation than coding region methylation (Fig. 2c). Conversely, genes that were identified as being heavily associated with RNA Pol-V showed the opposite pattern (Fig. 2C,D).

Perhaps less expectedly, we also found that different classes of genes varied in terms of the extent to which their methylation shifted in response to zebularine treatment (Zebularine Concentration * Gene Category: *p* < 0.005 in all contexts, Fig. 2C). The largest factor in determining the extent to which a gene was demethylated was it's baseline methylation in the control group, where, perhaps unsurprisingly, genes with higher starting methylation showed more extreme decreases in response to zebularine treatment (p < 0.0001, Fig. 2C).  While Zebularine is known to function as a cytidine analog that limits the activity of methyltransferases through decreasing disassociation between DMNTs and DNA^32^, this is the first piece of evidence that its impact on methylation across the genome varies across different classes of genes. Even after accounting for the impact of baseline methylation on the methylation shift in response to zebularine, we observe substantially larger decreases in CHG methylation signatures 4kb-2kb upstream of genes with TE-like methylation than expected by chance (p < 0.05) (Fig. 2c). Interestingly, these same genes showed subtle increases in gene body CHH and CHG methylation, suggesting a potential compensatory role of this form of methylation to limit transposon activation.

### Zebularine decreases mean methylation but increases variability

**Figure 3:**
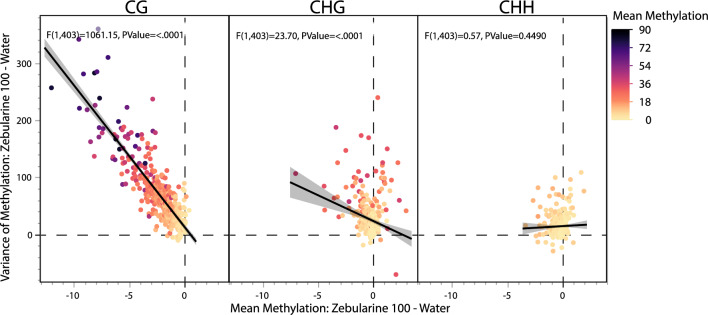
Zebularine induced decreases in CG and CHG but not CHH methylation are associated with concomitant increases in variance. After calculating mean shifts and variance shifts in the average methylation of the 5 kb region up-stream of our 403 genes with the most consistent read depths we ran linear regressions to look for associations between these two. We found that indeed genes with larger decreases in methylation in response to zebularine also tended to have much more variable CG methylation, and somewhat more variable CHG methylation. There was not a significant association between these two for CHH methylation.

While the above results focused on average methylation levels, and their shifts in response to zebularine treatments, the ability to multiplex samples on individual nanopore runs also allows for high enough sample sizes to study patterns of epigenetic variation within populations at reasonable costs. We found that in all three cytosine contexts, more highly methylated gene regions were also more variably methylated within any of our five treatment groups. For CG methylation, this correlation between mean and variance of the zebularine effect was stronger for cytosines in upstream (R^2^ = 0.575) than gene body regions (0.431). While the above results have demonstrated that highly methylated loci show the strongest methylation decreases in response to zebularine, and that highly methylated genes are also the most variably methylated, we also wanted to test if the cytosines that show the strongest decreases in mean methylation are the same ones that show increases in epigenetic variability. Indeed, for CG methylation we found a strong negative relationship between methylation shift in the mean methylation in the zebularine 100 µM treatment relative to water controls with a parallel shift in variance (Fig. 3). This pattern is less strong for CHG methylation, and not present for CHH.

These results confirm that not only does zebularine preferentially demethylate highly methylated regions, but also that these same regions have increased variability in zebularine treated plants. To test the extent to which epigenetic variation within population was due to global or region specific epigenetic variance, we compared residuals of unexplained methylation variance after accounting for individual to individual variation. We found that while zebularine treated plants had more residual epigenetic variance (mean absolute residual methylation: zebularine 25 µM: 11.91, zebularine 50 µM: 12.38, zebularine 100 µM: 12.28) than water (10.62), residuals were actually highest in the DMSO group (14.13). Thus, the treatment of zebularine led to stochastic demethylation across the genome leading to more mosaic methylation patterns within these treated populations than the water control group, but future work investigating the induction of mosaic methylomes in DMSO treated plants with and without zebularine will be necessary to help decipher a possible mechanism responsible for this pattern.

While gene-to-gene levels of methylation broken down by promoter regions and coding regions demonstrates methylation variation on a coarse scale, previous WGBS sequencing has demonstrated that the genome is mainly segregated into regions of very high and very low CG methylation, with some moderately methylated regions interspersed throughout. One way to detect these “methyl-regions” is through changepoint analysis using the “PELT” algorithm^33^ which provides a time efficient means to scan across the genome and identify regions where there is a mean shift in methylation after accounting for a manually specified penalty against over-fitting. Using “PELT” changepoint analysis we are able to identify the same CG epigenetic patterning as previously reported, with small to large regions with little to no methylation punctuated by small regions of moderate methylation, and small to moderate regions of very high (over 70%) methylation^33^. Non-CG methylation patterns, while generally much lower and lacking large regions with extremely high methylation, were similar in the presence of large unmethylated regions and small to moderate regions with higher methylation.

### Mixed-modeling approaches detect zebularine-induced changes in methylation

During the above analyses we detected not only significant methylome variability due to treatment, but also within treatment groups between individuals, and that this within-group variation appears to be treatment and context dependent. For this reason, it was vital to make our package not only robust to handling within-treatment group variation through a mixed model approach (this section), but also allow it to analyze individual specific DMRs relative to a control population (*Individual-based modeling identifies unique epigenetic responses to zebularine)*, as well as include in the output optional columns that track within treatment group variance for specific cytosines.

The R package presented here provides an easy framework to use standard R modeling formats using either binomial or beta-binomial mixed modeling to calculate per-cytosine summary statistics of the evidence of differential methylation (Note S2 and S3, Data S2). We ran three group-model variants to assess (1) differences in methylation between water and DMSO groups, (2) the shift in methylation between our Zebularine 100 um group and our DMSO and water controls, and a third, more complex model (3) that had fixed effects of both round and zebularine concentration. Within each of these models we recorded the Z-statistic, effect size estimate, and error of this estimate for every cytosine. Additionally, we allowed for the implementation of q-value estimation to account for issues of multiple testing. For model 3, we included random effects of individual and fixed effects of zebularine concentration and treatment round to identify regions of the genome that show dose sensitive responses to zebularine concentration. We found there to be a substantial overabundance of very low p-values associated with the zebularine effect leading to a substantial number of cytosines with q-values < 0.1 (n = 14,600 cytosines, Fig. S3), with far fewer sites showing significant differences between treatment rounds (n = 1839, Fig. S3). As expected, we found that Z-statistics for zebularine concentration tend to be negative (*cytosine contex: mean, sd, n*; CG: − 0.77, 1.18, 106,642; CHG: − 0.229, 1.167, 46,337; CHH: − 0.344, 0.97, 154,018), and are positively correlated with the change in methylation between zebularine and control treated plants (R^2^ = 0.399, *p* < 0.0001). Additionally, we found that mean methylation variance within treatment groups is negatively associated with the absolute value of Z-statistics (r = − 0.56). This makes intuitive sense, and in cases where a user is interested in identifying a region that shows *consistent* shifts in methylation, this result is desirable. However, as we previously found that zebularine treatment increases epigenetic variance, these highly variable sites may be of interest, and require individual based models to fully explore.

**Figure 4:**
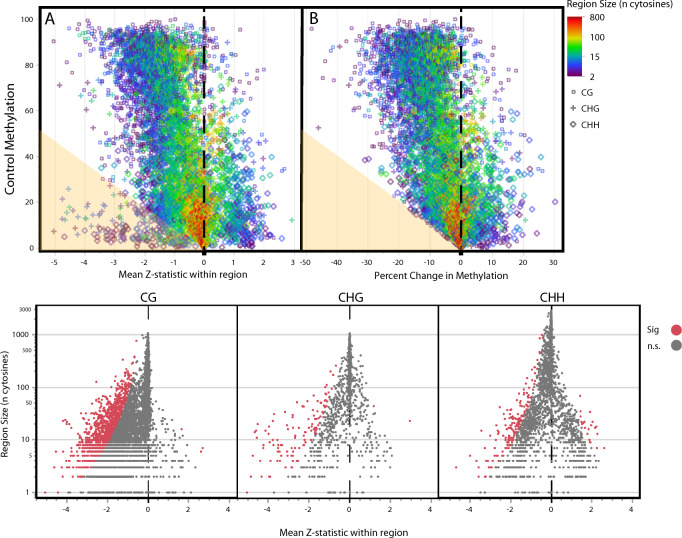
Changepoint analysis identifies regions of the genome where zebularine induced substantial shifts in methylation. (**A**) The large majority of changepoint regions have negative Z-statistics reflecting regions with consistent evidence of zebularine induced decreases in methylation, especially regions with higher baseline methylation. The strongest negative Z-statistics tend to be associated with very highly methylated CG regions as well as a fair number of CHG and CHH regions with relatively low starting methylation in control conditions (points below angled line). (**B**) When looking at raw changes in mean methylation patterns are very similar, however note that natural inability to confidently detect lowly methylated regions with strong decreases in methylation (lack of points below angled line). (**C**) Volcano plots showing the mean Z-statistic for a zebularine effect on methylation within a changepoint identified region vs. the size of the region. Red points are those that passed the significant DMR threshold.

Our approach first estimates the evidence of differential methylation for each cytosine independently, however in many cases it is contiguous regions of differentially methylated cytosines that have the strongest biological effect and are of most interest. To analyze these regional methylation differences, we once again used changepoint detection, however this time we used Z-statistics for methylation change, rather than baseline methylation, to find similarly altered regions of the genome. In total, we identified 6031 CG, 1442 CHG, and 2947 CHH contiguous regions of similar shifts in response to zebularine across our 426 genes. Cytosines in the CG context averaged 62 cytosines in length, significantly smaller than CHG (191) and CHH (347) cytosines. As identifying DMRs is often the main focus of methylation analyses, we next compared the genetic regions identified based on Z-statistics using this mixed-modeling approach, with a less rigorous changepoint approach based on mean shifts in methylation. While the results are largely concordant, we did find a small, but significant number of genomic regions with low baseline methylation (< 20%, CHG and CHH primarily) that showed highly significant decreases in methylation (based on Z-statistics), that would be easily overlooked looking only at mean shifts in methylation (Fig. 4). Assigning scores to DMR regions is somewhat arbitrary, but can also be useful in detecting regions with the highest probability of affecting phenotypic change. We developed a score (*SR*) accounting for mean Z-statistic within a given changepoint region, as well as the size of that region and set a threshold for our purposes equivalent to a single cytosine with an absolute Z-statistic of greater than 4 (*p* = 0.00063), a small region of 8 consecutive cytosines with a mean Z over 2, or a larger 64 cytosine region with an absolute Z of greater than 1. Of the 10,420 total regions, this approach identified 1793 that passed this significance threshold (17.2%) compared to only ~ 6.56 (0.063%) expected by random chance, with all but 26 having significantly lower methylation as zebularine concentrations increased. Using simulation, we found that power to detect a true DMR with a 60% change in methylation at this threshold was 0.8075, while the false positive rate was 0.0025. CG regions accounted for the majority of these significant DMRs (1294), however there were a substantial number of moderate and lowly methylated CHG and CHH regions that also passed this stringent threshold.

### Individual-based modeling identifies unique epigenetic responses to zebularine

**Figure 5:**
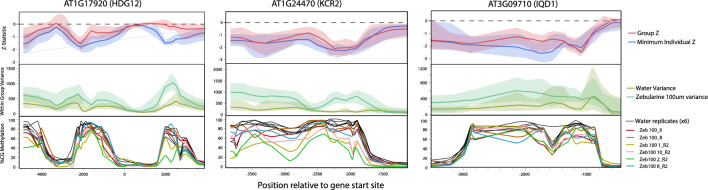
Test and summary statistics are both useful in identifying plant specific and consistent group shifts in methylation. Local kernel regression is used as a smoothing function to plot variation for Z-statistics (top), within group variances (middle), and individual methylomes (bottom). Regions where group Z-statistic confidence intervals are below 0 have high confidence decreases in methylation in response to zebularine treatment. While variance across the whole genome is elevated in the Zeb_100 treatment, there is substantial spatial variation for these patterns. In areas with statistically significant Z-statistics it may be useful to visualize individual methylomes to identify specific plants that show intriguing methylome responses. For instance, individual Zeb100_8_R2 shows substantial decreases in methylation relative to controls between 1.8 and 2.3 kb into the gene body of *HDG12*, and 3–1.5 kb upstream of *KCR2*.

While the above approach offers a flexible way to run differential methylation analyses in a mixed modeling framework, we also wanted to provide the ability to compare individuals of interest against control populations in a simple framework. As simulations demonstrated that unlike group versus group comparisons, beta-binomial models performed significantly better than binomial models when one level in a group contained only a single individual (Note S3, Fig. S4, Data S3), we use this approach when modeling individual shifts in methylation relative to a set of controls. We ran a model comparing methylation for each of our zebularine 100 µM individuals versus the full set of controls, in turn yielding 6 Z-statistics per cytosine. As expected, the mean of these Z-statistics was highly correlated with the group effect Z-statistics (R^2^ = 0.74), and cytosines with highly variable methylation in our zebularine 100 treatment group had larger spreads of individual z-statistics, with some individuals showing highly significant knockdown, and other little knockdown at all. While in our case we had no a priori hypotheses regarding specific genes of interest, it is often the case that one may be interested in identifying which individuals show the strongest methylation shifts around certain specific genes of interest. We identified three genes (*HDG12*, *IQD1*, and *KCR2*) with moderate to high levels of variance between treated individual Z-statistics to manually inspect shifts in methylation around these genes, and confirmed visually that global patterns were not due to abnormalities in the data (Fig. 5). These genes highlight that variance is higher in the zebularine treated group, and where variance spikes (about 2 kb into *HDG12* gene body), we see divergence between group and individual methylation models and individual zebularine treated plants that show striking differences not only relative to the water controls, but also to other zebularine treated individuals.

## Discussion

The ability to accurately and rapidly detect DNA methylation has broad implications; from applied purposes such as epigenetically-assisted plant breeding to foundational questions surrounding phenotypic plasticity and development. As discussed above, WGBS studies have been, and will continue to be, a major technology enabling these studies, but the advent of long-read sequencing with intrinsic methylome status encoded in the sequencing output will facilitate methylome analysis without the need for separate library preparations and across repetitive regions of the genomes. Recent improvements and developments in nanopore sequencing and analysis have made methylome characterization in complex genomes theoretically feasible and affordable. However, to date few pipelines have been developed to enable straightforward differential methylation analyses from this platform. Here, we demonstrate that relatively large differential methylation experiments (32 plants) can be performed affordably and with the accuracy and power to confirm previously identified effects of zebularine on methylation^30^, as well as detect novel patterns associated with this response. The most thorough study to date into the impact of zebularine on global methylation in *Arabidopsis* was done using WGBS and uncovered a number of interesting features around the loci impacted by these compounds^30^. Griffin et al*.* found that transposon expression increased in response to demethylation, methylation was reduced in the promoters of a number of evolutionarily important genes (*FWA* and *SDC*) in ways that reduced expression, and that these responses were concentration dependent. Here, we expanded upon this knowledge by using a barcoded rather than pooled sequencing approach, and focusing on 426 genes (Table S2) rather than the whole genome, in order to achieve sufficient read depths without excessive costs.

Looking at general patterns in our control versus treated groups, we found clear evidence of decreases in mean CG, CHG, and CHH methylation in line with previously reported results from WGBS. Additionally, we reaffirmed that it is very highly methylated cytosines that show the strongest decreases in methylation after zebularine treatment, and that CHG methylation shows a minimal decrease in methylation going from 50 to 100 µM, while the other two sequence contexts continue to decline. As this previous study pooled samples prior to library preparation, within group variance was not something that they could explore. Here, we find that the same highly methylated cytosines that have the strongest decreases in mean methylation, also have the strongest increases for within treatment group variability. This result presents the possibility that zebularine may not only be useful in generating demethylated plant lineages, but also in inducing epigenetic variability that could be useful in breeding or research efforts.

By integrating all methylome analysis steps after the creation of “*bedMethyl*” files into a single R package, we enable users to leverage the flexibility of mixed modeling within R to assess differential methylation, estimate effect sizes and errors, as well as Z-statistics, *p* values, and q-values on a per-cytosine level. Here, we focused primarily on the results of a model that examined the association between the zebularine concentration that a given plant was exposed to with its methylation level. While these individual cytosine Z-statistics may be of interest to some researchers, we also integrate a changepoint approach to enable the detection of groups of cytosines with similar shifts in methylation. This changepoint analysis can be run on any test statistic or summary statistic within *sounDMR*, and while we envision the primary use case would be changepoint analysis on Z-statistics, there is flexibility provided to look for regions with similar baseline methylation, or variability within groups. From these analyses it became clear that, while highly methylated regions did on average show the strongest decreases in methylation, there were also hundreds of lowly methylated CHG and CHH regions with highly significant decreases in methylation in response to zebularine. After identifying these regions, being able to assign *p* values or other scores or ranks to a given DMR is often useful in order to narrow down focus to specific regions that may have biological significance. While there are a number of approaches available to aggregate consecutive *p* values^34^, the combination of using test statistics that contribute to *p* values in the identification of a given region, along with complications associated with the lack of independence of methylation calls across nearby cytosines, makes any *p* value associated with these changepoint identified regions dubious. As an alternative, we developed a score that integrates the size of a DMR and the mean Z-statistic of the cytosines within it (*see Methods Data analysis*). The top DMR identified through this approach was a region of 23 CG cytosines in the region up-stream of *AT1G25054* with a mean Z-statistic of 4.19 and a mean shift of methylation of 30% between controls and zebularine treated plants. While any approach to score differential methylation will be somewhat arbitrary, we felt that this approach balanced the size of a differentially methylated region and confidence in the shift in methylation in a way that will maximize scores for DMRs more likely to affect biological change. Through the use of simulation we confirmed that the false positive rates for these mixed models were low (within 0.01 of alpha), and that for a range of sample sizes the power to detect a 70% change of methylation at a single cytosine with our mixed binomial model was between 0.78 and 0.94 (Note S3). One important caveat is that while false positives are not a large problem in our zebularine experiment, as the fraction of the genome differentially methylated by the treatment is quite high, in many cases researchers will be interested in scenarios where DMRs are few and far between. To allow scientists to test if specific regions show strong evidence of differential methylation relative to the genomic background we have implemented a bootstrapping approach to compare the strength of evidence for a DMR around a specific gene of interest relative to the genomic background.

Detecting significant changes in methylation between groups of individuals is the primary focus for the majority of methylome analysis, however there are many cases where the study of unique individuals is valuable. Here, we demonstrate that there is substantial epigenetic variability across our sequenced samples, and that this variance increases in response to zebularine treatment, providing evidence that environmental factors may not just be capable of changing mean methylation, but also its stochasticity. The induction of spatially variable epigenetic markings could prove useful in the generation of populations that function like epigenetic recombinant inbred lines (“epiRILs”) for foundational research or applied scenarios such as plant breeding. As genome sequencing technology advances and the detection of nucleic acid modifications becomes a more standard part of sequencing, methods such as *sounDMR* will enable meaningful examination of epigenetic variation across larger populations. The methods presented here will enable a broader adoption of low cost, high resolution epigenetic profiling in both plant breeding and foundational research.

## Methods

### Plant material, growth conditions, and zebularine treatments

*Arabidopsis* seeds of the Col-0 ecotype were acquired from the laboratory of Jacob Brunkard from University of California, Berkeley complying with all guidelines and legislation. *A. thaliana* seeds were sterilized using a 70% ethanol wash for 15 min and rinsed three times with sterile water before plating on half-strength Murashige and Skoog (MS) media (Sigma Aldrich; M3671) with 1 g/L 2-(N-Morpholino)ethane-sulfonic acid (MES) (Caisson Laboratories; MSP01), and 0.75% Bacto Agar (BD; 214,010). Zebularine treatments were performed according to Griffin et al. 2016. Zebularine (Sigma Aldrich; Z4775) was dissolved in DMSO and incorporated into the MS media after autoclaving at final concentrations of 25, 50, and 100 µM alongside sterile water and DMSO controls. Plated seeds were stratified in the dark at 4 °C for 3 days before transferring to a 10 h day photoperiod at 120 µmol photons m^−2^ s^−1^, 21 °C. Seedlings were grown on treated plates for 7 days and then transplanted to soil (Sunshine Mix #4) for 4 weeks until all plants were at the 1.14 principle growth stage (Boyes et al., Plant Cell). After 4 weeks, 5–6 plants per treatment group were sampled, with each plant representing one individual, unpooled. For sampling, 100 mg leaf tissue from the youngest fully expanded leaves from 5 to 6 individuals was collected within 1 h and frozen for DNA extractions. To achieve 100 mg of tissue, 3 of the youngest fully expanded leaves were collected for water, DMSO, 25 µM, and 50 µM zebularine treatment groups. For the 100 µM zebularine plants, 6 of the youngest fully expanded leaves were collected, as the leaf size was stunted despite being in the same developmental stage.

### DNA extraction, library preparation, and Oxford nanopore sequencing

Frozen leaf tissues were ground to a fine powder in a Geno/Grinder. DNA extraction was done using two kits. For the first batch of extractions, the Qiagen DNEasy^®^ Plant Pro Kit was used following the manufacturer's protocol with some modifications: homogenization in the Geno/Grinder was done before and after the addition of buffer CD1; samples were incubated on a Hula mixer for 10 min after homogenization; centrifugation at steps 3 and 6 were done at 16,000 × g; samples were eluted by adding 50 µL nuclease-free H_2_O that had been heated to 65 °C and incubated incubating for 4 min at room temperature prior to elution. In order to improve DNA purity, samples for the first round of sequencing were then treated with the Zymo Research Genomic DNA Clean & Concentrator™-25 kit, following the manufacturer’s protocol for large fragments. The quantity of DNA was measured using the Qubit Broad Range Kit (Invitrogen), while estimates of purity (260/280 and 260/230 ratios) was measured using a Nanodrop. One microliter of the eluted DNA was also loaded in the gel to evaluate DNA integrity/fragment size.

Multiplexed libraries were prepared using the ONT SQK-LSK109 and EXP-NBD104 kits with the following modifications to the manufacturer’s protocol: the genomic DNA input was increased from 1 to 2 µg; during repair and end prep, samples were incubated at 20 °C for 10 min, and at 65 °C for 10 min, and the volume of Ampure XP beads was reduced to 30 µL; during the barcoding and adapter ligation steps, reactions were incubated at RT for 20 min; and before final elution, samples were incubated at 37 °C for 20 min. Additionally, wide-bore tips were used for sample transfers at volumes > 20 µL in order to preserve larger fragments. Due to low individual sample quantifications, multiplexed pools consisted of five individuals each.

For the second round of sequencing, genomic DNA was extracted using Nucleospin Plant II Mini Kit with PL2 buffer (Macherey Nagel) following the manufacturer’s protocol. After testing, we found that this kit has better performance for Arabidopsis in terms of quantity and quality of genomic DNA compared to the Qiagen Plant Pro Kit. To maximize recovery of genomic DNA, elution was done twice, putting the eluate back to the same spin column and incubating at 65 °C celsius for 5 min both times. DNA quantity and quality were also evaluated using Qubit, Nanodrop, and gel electrophoresis. The same library preparation protocol used in the first batch of sequencing was used for Oxford Nanopore Sequencing (SQK-LSK109 and EXP-NBD104). In an effort to improve depth of coverage for each sample, the number of samples in each multiplexed pool was reduced to 3–4 instead of 5. A summary of how the samples were multiplexed, and which libraries were sequenced a second time, can be found in Table S1. In total, 32 samples were sequenced, consisting of 12 controls (6 water and 6 DMSO) and 20 zebularine treated (6 Zeb100 and 7 of each Zeb25 and Zeb50) individuals.

Finished libraries were loaded onto ONT flow cells R9.4.1 (FLO-MIN106D) slotted in a GridION sequencer. The flow cells were checked prior to the run and settings were configured using the *MinKNOW* software. To perform adaptive sampling, 425 genes were chosen from the Arabidopsis genome (TAIR10) that represent a wide range of functions and are expected to vary in their methylation patterns. PolV CHIP-seq and RIP-seq, AGO4 RIP-seq and siRNA data were downloaded from NCBI SRA database. Raw reads were trimmed using Trim Galore^35^, and trimmed reads were mapped using STAR^36^ or Shortstack^37^ with options “–nohp –mmap f”. Differential expression analysis between mock and PolV or AGO4-bound RIP-seq samples was performed using edgeR^38^, and genes enriched were selected for adaptive sampling. Genes were sorted according to siRNA abundance, and genes with high siRNA abundance were also included. These genes are also distributed throughout the five Arabidopsis chromosomes. In addition to the gene sequence, 10 kb of sequence upstream and downstream of the start and stop codons were included. The list of all the gene IDs used and their ± 10 kb genomic coordinates are listed in Table S2. A bed file (see sounDMR github) of these sequences were created and used as a reference to enrich for the targets. For adaptive sampling, the “fast” basecalling (dna_r9.4.1_450bps_fast) option was used with minimum Q-score of 8. Sequencing duration was set for 72 h, after which the sequencing data were transferred to a computing node with an NVDA RTX A4000 and Intel Xeon silver 4107.

### Data analysis

Basecalling and methylation calling were done concurrently using either ONT *Megalodon* with the 5 mC all-context R9.1 model, or using a combination of ONT-tombo and *DeepSignal-plant*. While it is relatively straightforward to generate these per-sample methylation files, converting this data into an appropriate file format for downstream analyses was not readily available, and led to us developing a pipeline to subset bedMethyl files to adaptive sampling regions of interest; merge individual bedMethyl files to create a combined experiment file; orient bases to the start sites of genes of interest; calculate summary statistics of methylation; run mixed models to generate strengths of evidence of differential methylation; perform changepoint analysis to identify methyl-regions; and finally output this data in a format that can be easily plotted within R, JMP, or elsewhere. This package as well as documentation can be found at https://github.com/SoundAg/sounDMR.

Briefly, this pipeline consists of six major commands. (1) generate_methylframe: takes in individual methylome data (bedMethyl) and creates a Methylframe (combination of all bedMethyl files into a single large data frame); (2) create_dmr_obj: takes in this data is to clean and combine into a single object; (3) create_methyl_summary: creates the methyl_summary frame to store the summary statistics; (4) find_DMR: runs mixed effects models where the user can select whether to run individual or group analysis using either a binomial or beta-binomial distribution where i the Individual is used for the random effect and the treatment group is used for the mixed effect.Within this model, Z-statistics is computed which is converted to *p* values, and then q-values using an R package (“*qvalue*”)^39^. The power comparisons between these two approaches is documented in Note S3; (5) changepoint_analysis: identifies changepoints in methylation based on the Z-statistics calculated in the previous step, where the user chose the PELT penalty values for each cytosine context; and finally (6) DMR_score: runs bootstrapping to identify differentially methylated regions close to and 10 kb away from the target gene and then renders a score for each cytosine context along with *p* values. Once these steps were completed, the output data frame was exported as a .csv file and loaded into JMP 16 for downstream analyses.

In order to compare DMRs we developed a score (*SR*) that encompasses the size of the region and the strength of evidence of differential methylation within the region. $SR={Z}_{mean}\sqrt[3]{n}$ where ${Z}_{mean}$ is the mean Z statistic within a given changepoint identified region and $n$ is the number of cytosines included in the region.

### Supplementary Information

Supplementary Information 1.

Supplementary Information 2.

Supplementary Information 3.

Supplementary Information 4.

Supplementary Table S1.

Supplementary Table S2.

## Data Availability

The raw sequence data generated in this study are available at Sequence Read Archive (SRA, https://www.ncbi.nlm.nih.gov/sra) under the BioProject ID PRJNA942940, accession numbers: SAMN33700246–SAMN33700277.
