# Supplementary material for: A tool for rapid, automated characterization of population epigenomics in plants
Source: Sci Rep. 2023 Aug 17;13:12915. doi: 10.1038/s41598-023-38356-7 (PMC10435466; doi:10.1038/s41598-023-38356-7)
Supplement: Supplementary file 4 — Supplementary Information 4. [file 41598_2023_38356_MOESM4_ESM.pdf]

Supplementary Figures: S1-S4

Supplementary Notes: S1-S3

Uploaded separate:

Supplementary Tables: S1-S2

Supplementary Data S1-S3

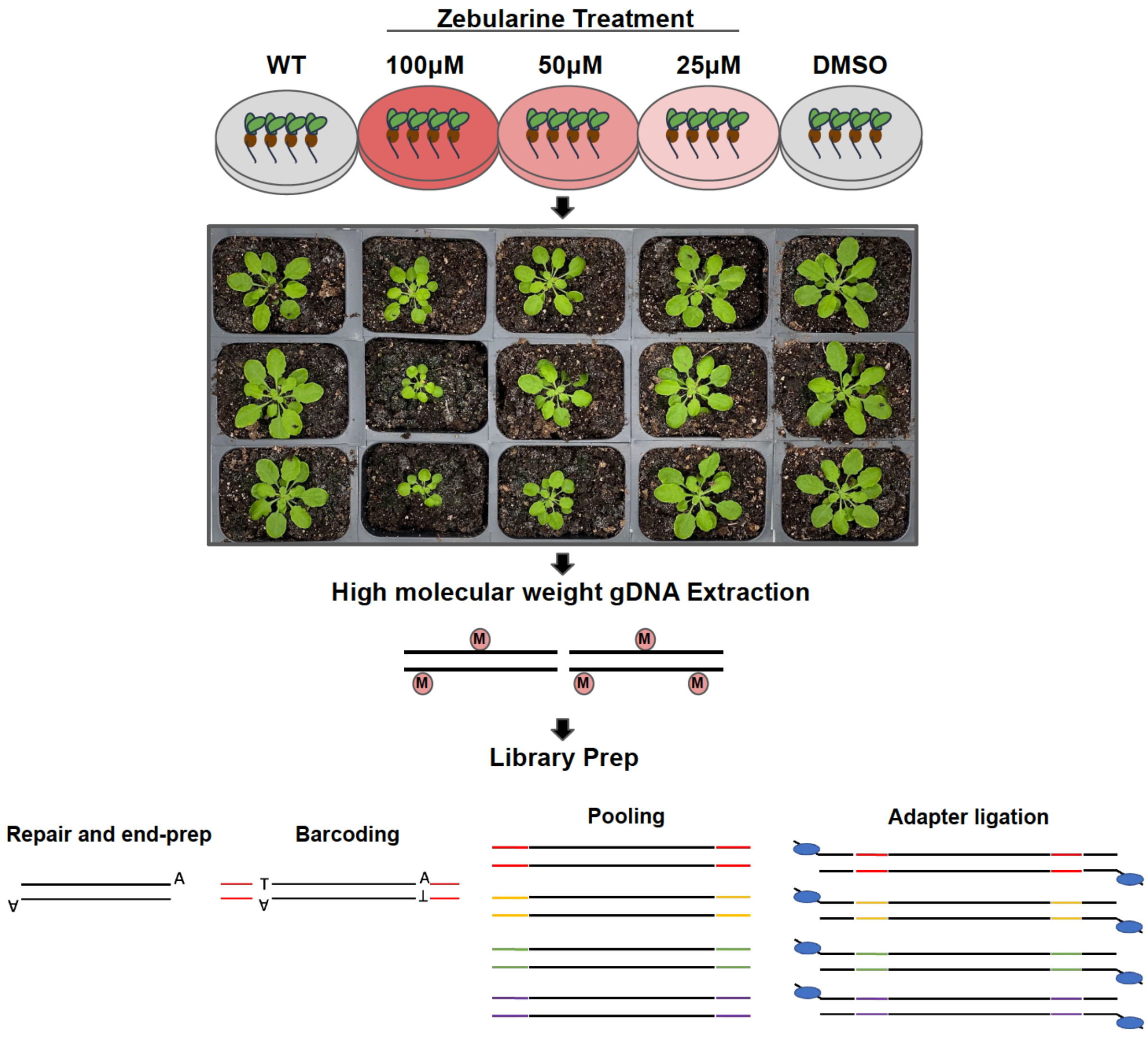

Figure S1: Experimental design showing effects of zebularine treatment through library preparation.

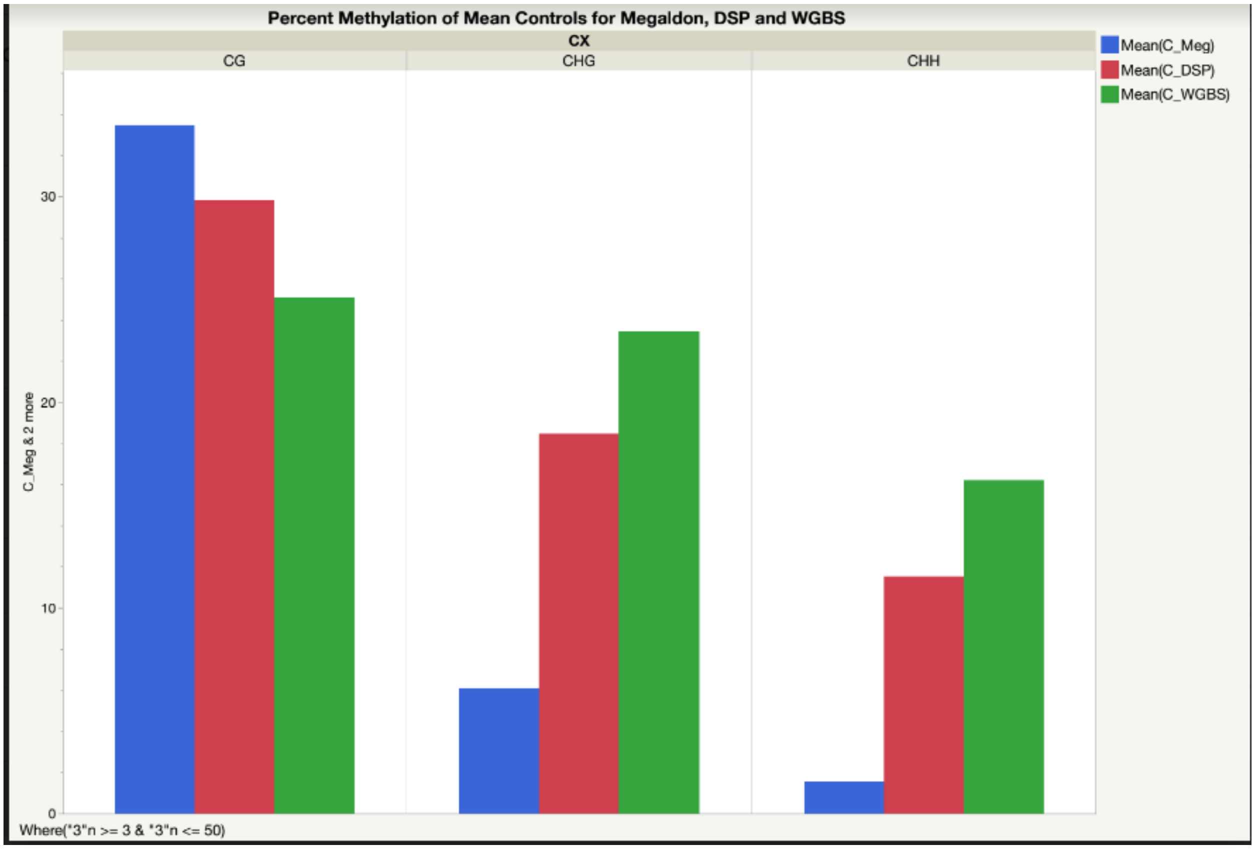

Figure S2: Mean methylation levels across cytosines with > 0 methylation in WGBS data (green) show higher concordance with DSP (red) than *Megalodon* (blue).

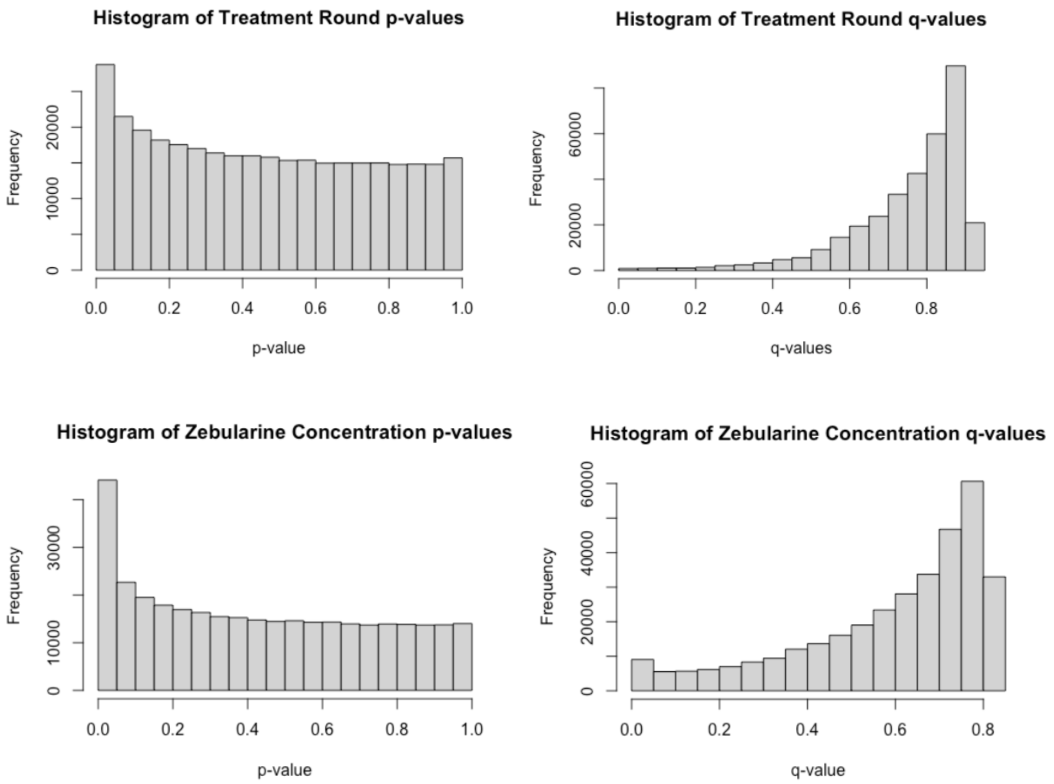

Figure S3: Distributions of p and q values from model considering fixed effects of zebularine concentration and treatment round.

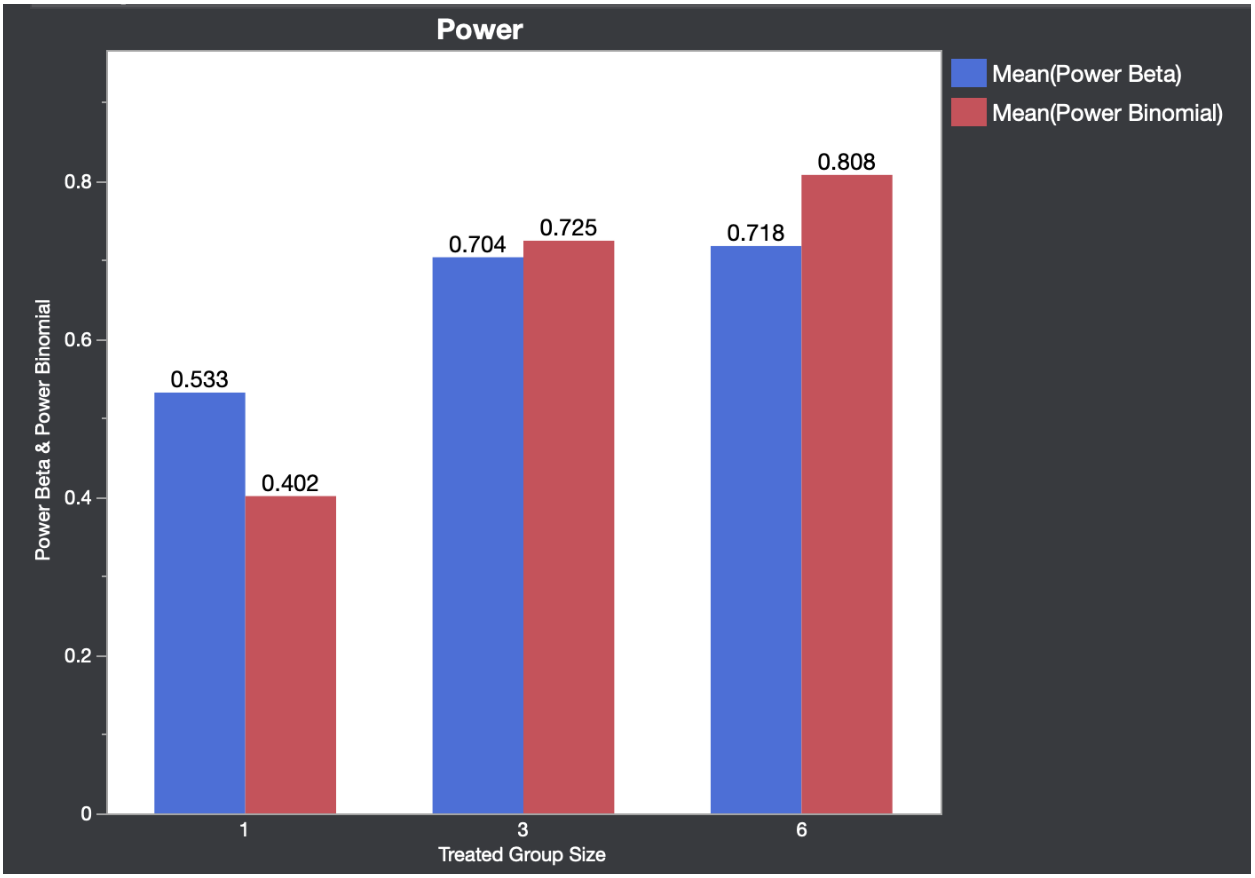

Figure S4: Measures of power (proportion of cytosines correctly called as differentially methylated at $p<0.05$ when true difference of 30% - false positives) for different treatment group sizes. Red bars: Binomial model, Blue bars: Beta-binomial.

Supplementary Note 1: Comparison of Deepsignal-plant and megalodon

In order to decide whether to use *Deep-Signal Plant* (DSP) or *Megalodon* methylation calls, we compared both of these with publicly available Whole-Genome Bisulfite sequencing (WGBS) data. We chose two water controls and two treated individuals from the same zebularine concentration (Zeb_100). These individuals were sequenced on ONT and analyzed for methylation through *Megalodon* and *DeepSignal-Plant*. This data was then combined to analyze the differences in methylation calls between DSP and *Megalodon* for both controls and treated individuals. The WGBS data was preprocessed to include the Percent_Methylation column from the unMeth and Methylated columns. The WGBS data was then merged with DSP and *Megalodon* methyl bed files in R. After some more preprocessing steps the final data frame had methylation percentages from each of the 3 water controls from *Megalodon*, DSP and WGBS respectively. The data was then input into the *JMP* tool for analysis and visualizations.

Supplementary Note 2: Package overview

Overview

The *sounDMR* package can be found at https://github.com/SoundAg/sounDMR and contains the code required to run the analysis. It contains a few basic steps: cleaning the data, finding differentially methylated regions, and finding the changepoints in those differentially methylated regions.

Creating the methylframe

The first step is to create a megaframe using the generate_megaframe() which gets called into the generate_methylframe() function that combines bedmethyl files from all samples in the experimental design. To do this, all the bedmethyl output files from the *DeepSignal-Plant* are put in a directory and then the working directory in the R environment is set. Once the bedmethyl files from all samples are in the R environment, using the generate_methylframe() function, either megaframe or zoomframe gets created. This entirely depends on whether the user has gene information or is just looking at the methylation on a whole genome level. In the latter case, megaframe is generated whereas for the zoomframe additional columns such as Gene, Zoom_co that separates the gene region from the upstream and downstream, a Zeroth_pos column to include coordinates zeroed-in based on every gene and Target_info column to specify if the a particular gene is a target or not, are created. This helps subset based

on the genes of interest in the downstream analysis. It also creates an experimental design starter that will be used for DMR analysis. Within this function, the megaframe is inspected for missing data. Based on the *ggplot*, a threshold for filter is selected and input into the generate_methylframe() function. This parameter is set to 0 but can be changed based on the *ggplot* and re-run as required.

Cleaning The Data

This section requires the data to be in the correct format. There are two files required: one containing the methylation data for each individual and one containing the experimental design.

This section encompasses the steps required to put the input data into the correct format for the DMR analysis into one function called create_dmr_obj(). This function takes in the methylation input data (methylframe) and the experimental design (Exp_ID). It creates an object of 6 dataframes: ZoomFrame_filtered, LongPercent, LongMeth, LongUnMeth, experimental_id_df, and Inputpers.. The ZoomFrame gets sorted; LongPercent is the ZoomFrame_filtered pivoted to be longer that includes percent methylation for every individual at every individual cytosine; LongMeth and LongUnMeth are the ZoomFrame_filtered pivoted to be longer with counts of methylated reads and unmethylated reads respectively; experimental_id_df is the same as Exp_ID unless it has some inconsistencies which are corrected for in the function; Inputpers is a dataframe of the percent methylation columns with a new column for the Mean Methylation. The LongPercent, LongMeth, and LongUnMeth data frames are aggregated by plant so two individuals that are clones will be pooled together.

Then the methyl_summary dataframe is created containing additional information for the Zoomframe_filtered including the Zeroth_pos column and a column for each individual showing the methylation change between the control, the average read depth, and a placeholder column for calculating the z-score of these methylation differences. This frame is created with the help of the create_gene_percent_x() function which utilizes dcast() from the *reshape2* package.

Finally in this stage, the methyl_summary frame is filtered to only include columns within the individuals_of_interest variable. These can be all individuals in the experiment or a selection.

The workflow for this section looks as follows:

```
 #Import the Geneco file
Geneco <- read.table(file.choose(), header=TRUE, sep=",")

# Find the methyl bed files within the working directory
All_methyl_beds <- list.files(path=".",pattern="*.bed")

# Generate the methylframe
#without gene info
Methylframe <- generate_methylframe(methyl_bed_list=All_methyl_beds,
Sample_count = 0, Methyl_call_type="Dorado", filter_NAs = 0,
gene_info = FALSE, gene_coordinate_file = NA, Gene_column=NA,
target_info=FALSE, File_prefix="Sample")

#with gene info
Methylframe <- generate_methylframe(methyl_bed_list=All_methyl_beds,
Sample_count = 0,Methyl_call_type="Dorado", filter_NAs = 0,
gene_info = TRUE, gene_coordinate_file = Geneco,
Gene_column='Gene_Name',target_info=TRUE, File_prefix="Sample")

experimental_design_df <- read.table(file.choose(), header=TRUE,
sep=",")

# Create the dmr object
dmr_obj <- create_dmr_obj(Zoomframe, experimental_design_df)

# Create the methyl summary
methyl_summary <- create_methyl_summary(dmr_obj, control = 'C')

# Option to subset methyl_summary
individuals = unique(dmr_obj$experimental_design_df$Individual)
```

```
methyl_summary <- subset_methyl_summary(methyl_summary, individuals_to_keep = individuals)
```

Finding Differentially Methylated Regions

This section identifies the differentially methylated regions. These regions can be identified between groups or for an individual compared to a group. For this analysis we use a mixed effects model based on either a binomial or beta-binomial distribution. There is one function for this: find_DMR(). This takes a number of arguments: the methyl_summary, dmr_obj fixed effects, random effects, the reads_threshold, the model_type, analysis_type, and optional control parameter. The reads threshold refers to the minimum read depth per cytosine required to run the model.

This function will go through each row in the methyl_summary and create a longer format of the methylated and unmethylated reads. When running individual analysis the function will compare the methylated and unmethylated reads of each individual against the control group. If either of the long data contains fewer reads than the reads_threshold, the function moves on to the next row in methyl_summary. Otherwise it will run the selected model and save the output to methyl_summary.

The binomial model is run using the *glmer()* function from the *lme4* package. The model first tries the *bobyqa* optimizer function and if unable to converge tries *nelder_mead*. The beta-binomial model is run using the *glmmTMB()* function from the *glmmTMB* package. If the model is unable to converge on either of these optimizer functions it will skip to the next row. When running group analysis, there are three columns added to the methyl_summary: the Z statistic, estimate, and standard error. When running the individual analysis, the Z statistic column created in the create_output_frame() function for each individual is updated to reflect the results of the model. This function can be re-run multiple times for different fixed and random effects variables. The individual analysis will update the z statistic columns accordingly. The group analysis will continue to add columns for each run of the function.

The workflow for this section looks as follows:

```
# Group Model Example
methyl_summary <- find_DMR(methyl_summary, dmr_obj, fixed = c('Group'), random = c('Individual'), reads_threshold = 3,
```

```
control = 'C', model = 'binomial',
analysis_type = 'group')
```

```
# Individual Model Example
methyl_summary <- find_DMR(methyl_summary, dmr_obj, fixed = c('Group'), random = c('Individual'), reads_threshold = 5, control = 'C', model = 'beta-binomial', analysis_type = 'individual')
```

Finding Changepoints

Changepoints are found using the changepoint_analysis() function. This function takes in the methyl_summary, penalty values for CG, CHG, and CHH contexts, and the column upon which to run the changepoint analysis. The penalty values are used to determine the amount of variability allowed within a changepoint. This function will create three additional columns to the methyl_summary: one for the mean methylation within the region, the length of the region in total bases, and a unique index value for the region.

The changepoint function will subset the data into gene and cytosine context before finding the changepoint means using the *cpt.mean()* function with the PELT algorithm and a manual penalty value from the *changepoint* package. A figure is created to show the methylation values of each gene for each context with the changepoints. This figure can be used to make decisions for modifying the penalty values provided to the function. There is an optional parameter called save_plots which will save the changepoint figures in the working directory. The information collected on each changepoint is then added back to the methyl_summary frame.

The workflow for this section looks as follows:

```
 #The genes of interest for the experiment
target_genes <- unique(dmr_obj$ZoomFrame_filtered$Gene)

methyl_summary <- changepoint_analysis(methyl_summary, CG_penalty = 9, CHG_penalty = 4, CHH_penalty = 7, target_genes = target_genes, save_plots = F, z_col = "Z_GroupT_small")
```

Scoring DMRs and permutation testing to calculate relative p-values

Once methyl-regions are identified, the user may use the functions below to assign scores to these regions to reflect the magnitude of a DMR within a given region. While these scores are somewhat arbitrary, we include three options within our package by default, but also allow users to generate their own custom functions. The two included scoring metrics are:

1) $SR = Z_{mean}\sqrt[3]{n}$

2) $dmrscore = Z_{mean}\sqrt{|\Delta p_m|}\sqrt[3]{n}$

Within this paper we use *SR*, which is a measure purely of statistical evidence associated with a DMR. Z_{mean} is the mean Z test statistic for all of the cytosines within a region, while n is the number of cytosines within a region. The *dmrscore* function adds an additional scalar which is the magnitude of the change in methylation between the groups considered, $\sqrt{|\Delta p_m|}$.

Once these scores are assigned to each methyl-region, the user is given the option to compare the dmr-score of a specific region or gene of interest with the distribution of dmr-scores across the genome. This approach is specifically designed for cases where a user may have an *a priori* hypothesis as to whether or not a specific region of the genome is differentially methylated. Users can specify a specific gene of interest included in the geneco file, as well as the region around that gene of interest (relative to the gene start site). Additionally the user specifies the statistic to use (*SR* or *dmrscore*) and the number of permutations. The function looks for the strongest DMRs of each cytosine context around the target region designated and applies a decay function to the scoring of DMRs outside of the region included. Then the function sub-samples other regions of the genome that have sufficiently sized flanking regions and randomly samples these regions, applying the same scoring and decay function to assign scores to other regions in the genome. Finally, the bootscore function assigns p-values for the target region as well as adjusted dmrscores based on how the given region ranks relative to the genomic background.

```
DMR_score <- sound_score(changepoint_OF = methyl_summary,
```

```
                              Statistic = changepoint_cols[1],
                              Per_Change = "Treat_V_Control",
                                                  other_columns=c("Control",
"Estimate_GroupT_small"))


boot_score(sound_score_obj = DMR_score, target_gene = "AT1G01640",
scoring_col_name="dmr_score2")
```

Supplementary Note 3: Simulation of methylation data to analyze power and compare modeling approaches

Overview

DNA methylation data was simulated in R using a pipeline that involved three steps: (1) selecting parameters, (2) generating simulated methylation data, and (3) running binomial and beta-binomial random effects models to test for differential methylation in the simulated data. In total we tested over 50,000 different scenarios with 200 simulations per scenario.

Creating the Parameters

For these simulations there were two groups: A and B. For each group there were a number of adjustable parameters: group size, read depth, and percent methylation. The size of the group refers to the number of samples or individuals while the read depth refers to the coverage of DNA for that group. The percent methylation refers to the proportion of methylated reads for that group. Additionally, there was a shared noise variable which refers to the standard deviation of the normal curve surrounding the draws from the mean methylation percent.

The parameters used in this study included: group A size of 1, 2, 3, 4, 5, 6; group B size of 2, 3, 4, 5, 6; group A read depth of 4, 6, 8, 10, 12; group B read depth of 2, 3, 4, 6, 8, 10, 12; group A percent methylation of 0.2, 0.4, 0.6, 0.8; and noise variation of 0.1 and 0.2. Group B percent methylation values were chosen to be either identical to group A, or with a true methylation ranging from 10% to 70% lower than group A. This scenario reflects situations where either group A and B have equal methylation, or where group B has lower methylation ranging from very minor (10%) to quite large (70%).

Running the Simulations

To generate simulated methylation data, an R script takes in the parameters. For each combination of parameters, 200 rows of data were simulated to represent 200 unique cytosines. The columns include “count methylated” and “count unmethylated” for each individual for both groups. For example, with a group A size of 2 there would be 4 columns for group A: a1_meth, a1_unmeth, a2_meth, a2_unmeth. For each individual the read depth for that group was used to indicate the DNA coverage at that row (simulated cytosine). A read depth of 6 in the example above would mean that the count of methylated reads in a1_meth + the count of unmethylated reads in a1_unmeth would be equal to 6.

The percent methylation refers to the “true” group average for the proportion of methylation at a given site. Rather than simply drawing from a binomial model with that proportion (which would lead to higher confidence, due to lower dispersion), we developed an approach to induce individual to individual noise to the data to account for biological variation explicitly (along with technical variation which is accounted for by sampling error). To do this, percent methylation values were first arcsine transformed. Then we took random draws using *rtruncnorm(),* with this transformed value as the mean, the noise variable as the standard deviation, and limits of 0 to π/2. Finally these values were back transformed into probabilities by taking the sine of the draws squared.

Once the values for the probability of methylation were obtained for each row for a given individual, binomial draws were taken using *rbinom(),* with the probability being the percent methylation value, number of observations equal to 1, and the number of trials equal to the read depth of the group. The binomial distribution gave us the number of methylated reads. The number of unmethylated reads was calculated by subtracting the methylated reads from the read depth.

Testing Simulated Data

Once the data has been simulated, it is passed into another R script to run binomial (*glm()* from the *lme4* package) and beta-binomial (*glmmTMB()* from the *glmmTMB* package) random effects models using the same pipeline as within the *sounDMR* package to test for a difference in methylation between the two groups. These models were run for each row of the simulated data and the mean and median of the model statistics (p-value, estimate, error) were calculated. Finally the proportion of rows with a p-value below three alpha thresholds (0.1, 0.05,

and 0.01) were calculated. These summary statistics (mean p-value, median p-value, mean estimate, median estimate, mean error, proportion of rows < 0.1, proportion of rows < 0.05, and proportion of rows < 0.01) were added back to a data frame containing the simulation parameters. This allowed us to find the summary statistics for each model for a given set of parameters.

Results

The results of the simulations showed differences in power (true positive results at a true delta methylation of 0.3 - false positive methylation) between the binomial and beta-binomial models. The beta-binomial model showed stronger power when there was one individual being compared to a group, while the binomial model showed stronger power when comparing one group to another (Fig S3). We also compared the false positive rates between the two models and found similar results. With a true methylation change of 0, both models show a false discovery rate of about 0.098, 0.05, and 0.01 for p-values below 0.1, 0.05, and 0.01 respectively. In comparison, the true positive rates for these alpha thresholds, taken when the true difference in methylation is 70%, was found to be 0.94, 0.91, and 0.78 respectively in the binomial model and 0.84, 0.76, and 0.54 respectively in the beta binomial model. Additionally, as expected we see that in general as read depths and individuals per treatment group increase, so too does power.
